# Our Over-Crowded Profession

**Published:** 1912-11

**Authors:** 


### Our Over-Crowded Profession.

"The whole number of physicians in Buffalo is 78, including Homœopathic, Botanic, Thompsonian, Uroscopic et id omne genus. The number of *regular* practitioners (we cannot distinguish as *legal,* since the law now recognizes equally those of every order) is about one-half of the above number. Our population is a little short of 30,000. We have thus a professed practitioner for less than every four hundred. * * * that all cannot subsist on the necessities of disease is reduced to a mathematical demonstration."

The above is from the BUFFALO MEDICAL JOURNAL of 1845 (page 162.) At present, the list is 660 physicians, to about

420,000. As about 60 physicians are engaged in other business, moved away, dead or fully retired, the present ratio is about 1: 700. While the Homœopaths are now practically reunited with the "regular" practitioners and the other schools mentioned are dead issues, the osteopaths, optometrists, Christian Scientists, etc., bring the ratio about to its original basis. In addition, it must be remembered that there is far less disease than in 1845, much more paternalistic care of the sick and relatively insignificant demand for medical services in the outlying districts.

Our predecessor goes on to say that many physicians sought Buffalo as a promising western town and that, on account of disappointment, more than the existing number of physicians had moved away during a period of ten years. As we stated in the Aug., 1912, issue, only about a quarter of physicians practicing here in 1899 and recorded in a group picture, had departed, either to other places, other occupations or to a better world. In other words, the profession is much more stable and probably really better supported than 67 years ago. And, as did our distinguished predecessor, we are glad to welcome newcomers; however little the statistic need for them.

